# The National Insurance Act Department

**Published:** 1912-03-09

**Authors:** 


### NATIONAL INSURANCE ACT DEPARTMENT.

### Practical Help and Counsel.

In accordance with the announcement we made last week, we have the pleasure to explain the scope and intention of this new department of The Hospital. It is abundantly evident that a multitude of questions and many difficulties must arise in the minds of most persons who come within the scope of the National Insurance Act. We are therefore organising a special department which will be conducted by the Editor, with the aid of expert actuarial and other qualified assistants, so that practical help upon every point may be brought within the reach of all our readers. We shall naturally devote special attention to the working of the Act so far as it affects (*a*) members of the medical profession; (*b*) matrons, sisters, and nurses; and (*c*) male and female employees generally of voluntary hospitals and kindred institutions.

### How the System will Work.

The system adopted is very simple, for it consists merely in requiring that the coupon to be found on the third page of the cover of each issue shall accompany every communication, when information or help is sought from this department. We invite correspondence, suggestions, and inquiries upon all points. Already a number of people must have been placed in a position of doubt on various points in regard to how the Insurance Act will affect them or those dependent upon them. We offer a ready solution to every such person by writing at once to the Editor of this department. By this means all the employees of voluntary hospitals and kindred institutions, all matrons, sisters, and nurses, as well as members of the medical profession, may readily obtain, free of cost, all the help and information they may require now or in the future.

### Workers who Help the Voluntary Hospitals.

Of late years men and women workers of the artisan class have come forward with increasing generosity in support of the voluntary hospitals. Their position will be changed by the National Insurance Act, because the relations between the voluntary hospitals and the working classes may be revolutionised. Formerly working men and women gave to the voluntary hospitals, and they used them freely, with great advantage to themselves and the cordial co-operation of hospital managers, whenever they required in-patient or out-patient benefit.

### A Probable Revolution.

The National Insurance Act cuts the insured out of all out-patient benefit by providing them independently with what Mr. Lloyd George claims to be "adequate medical relief." For this medical relief both women and men among the workers will pay from 3d. to 4d. a week each, but they will cease to be eligible to receive free benefit from the hospitals. In regard to in-patient relief the National Insurance Act makes no provision, and, as we have often pointed out, if Mr. Lloyd George is correct, insured persons will clearly be ineligible to receive free in-patient relief, except as an act of grace, on the part of the hospitals, in exceptional cases. That is the real position, but the London voluntary hospitals have so far contented themselves with a declaration that they will wait and see how the Act works before deciding who will and who will not be eligible for free hospital benefit. Others have decided not to accept insured persons for free out-patient relief except when brought to the hospital by their medical attendant; whilst the question of in-patient relief is left an open one.

### Questions requiring Answers.

This being the position of affairs, it is certain that many questions will arise which will directly affect the interests of the working classes of both sexes, both in regard to the best way to secure the benefits of the Act, and the simplest means whereby every such worker can most readily procure the institutional aid he or she may be in need of when overtaken by severe illness. The working classes as a whole have nobly supported the voluntary hospitals in recent years, and we desire to place at their disposal in return, free of cost to them, all the help and information possible. With this in view we have made arrangements to meet the cases of artisans and workmen generally.

### How to Use the Department.

A perusal of the article which follows, giving the preliminary points of interest in regard to the insured which arise under the National Insurance Act, will indicate how important it is that insured persons shall have a ready means of submitting their difficulties to some authority which will deal with them sympathetically and endeavour to secure the maximum of benefit under the Act. One of the most important matters is the wise selection of an approved society of which they can become members, for by far the greater number of those who will be affected by the Act have yet to make a choice, and to equip themselves with knowledge whereby they may secure the maximum benefits each can claim. The field is a wide one, and we have therefore organised this department, and are prepared to help to the fullest possible extent all interested, who care to avail themselves of our services. All they are asked to do is to state their difficulties or wishes, enclosing them, with the coupon to be found on the third page of the cover, in an envelope addressed:—

INSURANCE.
The Editor, "The Hospital,"
29 Southampton Street, Strand, London, W.C.

